# 

**Published:** 1914-02-15

**Authors:** 


### ANNOUNCEMENTS.

The Southwestern Michigan Dental Association met at Battle Creek, November 17 to 19th, and elected the following officers for the ensuing year: President, S. J. Lewis of Kalamazoo; Vice-President, B. R. Parrish, of Battle Creek; Secretary-Treasurer, R. A. Bowie, Three Rivers. The meeting was well attended and the papers and clinics were of exceptionally high order. The entertainment was in keeping with the well known generous hospitality of the Breakfast Food City.

Officers, Arizona Dental Society, elected for the ensuing year are: President, W. A. Baker, Tucson; Vice-President, J. L. O'Connell, Phoenix; Secretary-Treasurer, H. H. Wilson, Phoenix. The meeting was held in Phoenix, November 5th to 7th, 1913.

The Kentucky State Dental Association will hold a four days post graduate course at the Seelbach Hotel, in Louisville March 9, 10, 11 and 12, 1914.

The lectures for the course are: Dr. Geo. H. Wilson, of Cleveland, "Prosthetic Dentistry," Dr. Thos. P. Hinman, of Atlanta, "Operative Dentistry," and Dr. A. J. Bush of Columbus, "Crown and Bridge Work."

We will also have papers and clinics for those who do not care to take the post graduate course.

The usual invitation is extended to all practitioners to attend.

We are already assured of a successful meeting.

Charles R. Schacklette, Sec'y.

### Prize Contest.

With the year 1914, begins the 30 anniversary of the Osterr-Ungar Vierteljahrsschrift fur Zahnheilkunde, Vienna,

(Austria) I. Petersplatz 7. In commemoration the editor offers three prizes as follows:

| | | |
|---|---|---|
| First Prize, | 1000 Kronen | or $200 |
| Second Prize | 600 Kronen | or 120 |
| Third Prize, | 400 Kronen | or 80 |

for scientific work on any branch of Dentistry theoretical or practical, but awarded according to its clinical value.

Herr Reg.-Rat Prof. Dr. Julius Scheff has accepted the chairmanship of the committee to judge and award prizes.

CONTEST REQUIREMENTS.

I. The contest is open to dentists of all countries. In case the work is in a foreign language a German translation is to be included with the application.

II. The work must be that which has not before appeared in print.

III. The work must be anonymously sent in bearing a certain word of identification with a sealed envelope, bearing the same word, containing the contestants name and address.

IV. The contest closes May 15th, 1914, as prizes are awarded July 15th, 1914.

V. The successful works will be published in the Osterr.-Ungar Vierteljahrsschrift fur Zahnheilkunde "when its size" permits.

VI. The editor claims the privilege to publish at the customary remuneration any of the works of the unsuccessful contestants.

JULIUS WEISS, Editor,
I. Petersplatz 7, Vienna, Austria.

### INDIANA STATE DENTAL ASSOCIATION.

Special Announcement. Meeting place has been changed to the German House. The 1914 meeting will be held in the German House. The prospects for a great meeting were so flattering, that after due consideration, the officers decided that it would be necessary to secure larger and more commodious quarters for this meeting, which we are planning to make equal to the "Semi-Centennial Jubilee Meeting."

The entire program will be given in the German House. Dr. Lucas, Master of Exhibitors, has already contracted for the building of booths for the exhibitors, and the decorating of the entire building.

The arrangement of the German House is ideal for a big meeting; fine auditorium for all sessions of the association, a great hall for night meetings, an ideal place for exhibitors, and the only place in Indianapolis that meets the requirements of a "Progressive Clinic." From every standpoint this 1914 meeting will excell any other meeting ever held in Indiana.

"A better individual dentist" for Indiana is our aim this year, and the officers have planned to reach that aim, but we must have your co-operation and presence, so mark of the dates NOW.

Wishing you a happy and prosperous year and hoping to meet you at this great meeting in May, I am,

Fraternally yours,

OTTO U. KING, Secretary.

### KENTUCKY STATE BOARD OF EXAMINERS.

At a meeting of the Kentucky State Board of Dental Examiners, held Oct. 9th, 1913, in Louisville, the following was adopted:

On and after January 1st, 1914, the Kentucky State Board of Dental Examiners will consider as reputable *only* such dental schools as conform strictly to the following rule:

1. That on and after January 1st, 1914, no dental college or dental department of a university shall accept for enrollment any student who shall not place on file with said dental college or dental department of a university, prior to such enrollment, credentials proving that he or she is a graduate of a high school or academy requiring a four years course of study, or possesses a general education equivalent to that required for graduation from a high school having a four years course of study beyond that of the elementary school.

2. The Kentucky State Board of Dental Examiners will examine graduates of reputable dental schools *only*.

3. Applicants for examination by the Kentucky State Board of Dental Examiners will be required to present to the Board a certified copy of the preliminary educational credentials on which he or she was admitted to the dental school at which he or she matriculated.

The Kentucky Board will rate as not reputable any dental school whose graduate shows on examination that his preliminary education was not up to the standard of the above rule.

KENTUCKY STATE BOARD OF DENTAL EXAMINERS,
J. H. BALDWIN, Secretary.

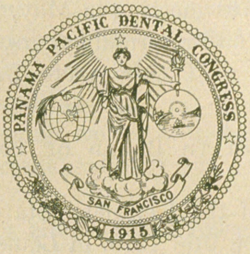

OPENS AUG. 30TH, 1915

### PANAMA-PACIFIC DENTAL CONGRESS TO BE BIG FEATURE OF THE PANAMA-PACIFIC INTERNATIONAL EXPOSITION

More than 3,000 dentists from every part of the civilized world now are expected to attend the Panama-Pacific Dental Congress to be held in San Francisco during the Panama-Pacific International Exposition, which will be open from February 20 to December 4, 1915, inclusive.

The Dental Congress, which is being promoted by prominent dentists of the Pacific Coast, promises to be the largest gathering of dentists ever held and will be the motif for discussions and addresses on the latest advances in dentistry. The Congress will convene August 30, 1915 and will remain in session for ten days.

A feature of the Congress will be a great clinic, at which the latest methods of dental surgery, practiced in every country of the world, will be demonstrated. It is expected that dentists of international reputation will attend the Congress and the discussions of the thousands of delegates will mark an epoch in the history of dentistry.

"Representatives of every country where dentistry is practiced will attend this Congress," said Dr. Frank L. Platt, of San Francisco, who is chairman of the local committee of organization. "It will be a thoroughly international gathering. Papers will be read on the most advanced subjects known to the profession and many of the participants will be men of international reputation. The latest methods used by dentists in various countries will be demonstrated in a great clinic, where there will be from 25 to 50 chairs, and all kinds of dental operations will be performed for the benefit of the thousands of assembled dentists."

The Dental Congress will be financed by a corporation known as the Pacific Dental Congress Commission of 1915. Active work toward securing international representation is in the hands of a committee of organizations, representing the dentists in the Pacific Coast States. This committee consists of a director in each of the states of Idaho, Washington, Oregon, Utah and Arizona, together with ten members of California. Dr. Platt is chairman of this committee and Dr. Arthur M. Flood, also of San Francisco, is secretary. Other members are Dr. F. G. Baird, San Francisco; Dr. H. A. Frederick, San Francisco; Dr. F. G. Jarvis, Oakland; Dr. Joseph Loran Pease, Oakland; Dr. H. G. Chappel, Oakland; Dr. R. B. Giffen, Sacramento; Dr. George F. Stiehl, Salt Lake City, Utah; Dr. B. M. Brookfield, Idaho Falls, Idaho; Dr. George T. Williams, Seattle, Wash.: Dr. Arthur W. Chance, Portland, Ore.; Dr. H. H. Wilson, Phoenix, Arizona; Dr. Charles M. Benbrook, Los Angeles; and Dr. T. Sydney Smith, Palo Alto, California.

Pacific coast dentists already have subscribed $13,000 for the promotion of the Congress and of this $7,000 has been collected to date. This money is being used in publicity work and invitations to the Congress are being sent out broadcast over the world. Executive committees are being appointed in every country where a dental organization exists.

A striking feature of the Congress will be the exhibits by dental manufacturers and dealers, which will include modern apparatus and appliances. As the Congress will meet in the new Municipal Auditorium, this array of interesting exhibits will be housed in the same building during the session of the Congress. Two thousand front feet of space will be occupied by the exhibits.

While these exhibits will be on display only during the Congress, there will be an extensive array of dental exhibits throughout the Exposition in the Palace of Liberal Arts, one of the eight main exhibit palaces. These will include apparatus and instruments used in dentistry, specimens of bridge and plate work, and the latest electrical devices used in dental surgery.

The modern application of electricity to dental uses, one of the most recent advances in dentistry, will receive important attention at the Dental Congress and much of the latest apparatus on display will illustrate this subject.

Other advances made by dentists of every country will be considered and especial attention will be given to scientific elimination of pain in dental work.

A number of national and state dental societies and fraternities will meet with the Panama-Pacific Dental Congress, instead of holding their individual annual meetings. Among those that already have signified their intention of meeting with the Congress are the National Dental Association and the American Society of Orthodontists.

To promote this great Congress of dentists ample time was essential to complete a working organization, and to start an effective campaign which would guarantee the carrying out of the extensive programme mapped out. It is expected the membership fee ($10.00) will pay the expenses of the Congress, but to meet the current expenses which are entailed in an affair of this magnitude it was decided to incorporate under the laws of California and issue Debenture Certificates to the total of three thousand bearing a face value of $10.00 each.

These certificates are now offered to the profession at large and may be paid for in two installments, full payment must be made by May 1, 1914. No promises can be made that dividends will be paid or that a return will be made of the money raised from the sale of these Debentures, but it is reasonable to suppose that after the expenses of the Congress are paid there will be funds sufficient in the Treasury to reimburse those who have been loyal and enterprising enough to subscribe and make this Congress possible.

We count on the loyalty of the West to the West, and at this greatest of opportunities to hold a Dental Congress in San Francisco when all the world will be coming to California at the official opening of the Panama Canal, it behooves our dentists to get in line and identify themselves with this Congress by buying one or more shares, and each and every one a membership. A membership is $10.00, and is separate and apart from the Debentures. Those wishing for Debentures or Memberships should address all communications to

ARTHUR M. FLOOD, Secretary,
240 Stockton Street,
San Francisco, California.

### THE SIXTH INTERNATIONAL DENTAL CONGRESS, LONDON, ENGLAND, AUGUST 3-8, 1914

The 6th International Dental Congress will be held in London, England, August 3-8, 1914. The committee appointed by the National Dental Association having in charge the affairs of the Congress relating to the U. S. A. have selected the following to take part in the Congress program:

### ADDRESSES.

Dr. H. J. Burkhart, Batavia, New York, to deliver the address on behalf of the National Dental Association at the opening session.

Dr. Edward C. Kirk, Philadelphia, Pa. Address before the general session, the afternoon session of the opening day, "The Tendencies in Dental Education."

### REPORTERS.

Section 1.—Dental Anatomy, Histology and Physiology. "The Evolution of the Human Dentition," Dr. I. N. Broomell Philadelphia, Pa., "Calcification," Dr. A. R. Starr, New York City, N. Y.; "Chemistry and Physiology of Saliva," Dr. Edward C. Kirk, Philadelphia, Pa.

Section 2.—Dental Pathology and Bacteriology. "The Etiology of Dental Caries," Dr. B. Holly Smith, Baltimore, Md.; "The Etiology and Pathology of Pyorrhea Alveolaris," Dr. Percy R. Howe, Boston, Mass.; "Pathological Conditions of the Dental Pulp," Dr. R. W.Bunting, Ann Arbor, Mich.; "The Pathology of the Antrum," Dr. Chas. H. Oakman, Detroit, Mich.

Section 3.—Dental Surgery and Therapeutics. "Inflammatory Disease of the Gigival Margin and Periodental Membrane (Pyorrhoea Alveolaris)," Dr. T. Sidney Smith, Palo Alto, Calif.; "Restorations of lost portion of tooth substance by Inlaying," Dr. R. Ottolengui, New York City, N. Y. "Oral Sepsis in relation to general disease," Dr. C. N. Johnson, Chicago, Ill.; "The prevention of Oral Sepsis by treatment," Dr. J. D. Patterson, Kansas City, Mo.

Section 4.—Dental Physics, Radiography and Metallurgy. "The uses and advantages of X-rays as an aid to diagnosis, including the differentiation of the radiography appearance of normal and abnormal tissue," Dr. Howard R. Raper, Indianapolis, Ind.; "The Structural and other changes in connection with Metals used in the Mouth," Dr. Clarence J. Grieves, Baltimore, Md.; "The Theory and

Practice of Pressure Casting," Dr. Weston A. Price, Cleveland, Ohio.

Section 5.—Dental Prosthesis. "Articulatio nand Articulators," Dr. J. H. Prothero, Chicago, Ill.; "Design and Retention of Partial Dentures," Dr. H. J. Goslee, Chicago, Ill.

Section 7.—Oral Surgery and Surgical Prosthesis. "The late results of cleft palate operations," Dr. Truman W. Brophy, Chicago, Ill.; The treatment of dental and dentigerous cysts," Dr. Wm. Carr, New York City, N. Y., "Surgical Prosthesis of the Jaws," Dr. M. C. Smith, Lynn, Mass.

Section 8.—Anaesthesia, General and Local. "Gas and Oxygen, alone in mixture and in sequence for the Extraction Operation," Dr. Chas. K. Teter, Cleveland, Ohio; "Gas and Oxygen Analgesia for conservative operations," Dr. Thos. B. Hartzell, Minneapolis, Minn.; "Local Anaesthesia with special reference to (*a*) Methods, (*b*) Drugs, (*c*) Sphere of usefulness, (*d*) Contra-indications and Dangers," Dr. Eugene R. Warner, Denver Colo.

Section 9.—Oral Hygiene, Public Instruction and Public Dental Service. "The Effects of Dental Treatment on National Health and Physique," Dr. Herbert L. Wheeler, New York City.; "Prophylaxis at different ages, "Dr. A. R. Melendy, Knoxville, Tenn.; "Lantern demonstration of slides showing (*a*) Means of affording public instruction in dental hygiene, e. g., lecture material, charts, etc.; (*b*) Photographs of School Dental Clinics, Institution for Public Dental Service for Adults or other Institutions in which public dental treatment is being carried out," Dr. Wm. A. White, Phelps, N. Y.

Section 10.— Dental Education. "Methods of Teaching Orthodontics to Dental Students," Dr. S. H. Guilford, Philadelphia, Pa.

The following have been selected as Honorary Presidents of the Section:

Section I. Dental Anatomy, Histology and Physiology. Dr. M. H. Cryer, Philadelphia.

Section II. Dental Pathology and Bacteriology, Dr. Thos. B. Hartzell, Minneaoplis.

Section III. Dental Surgery and Therapeutics, Dr. Edward S. Gaylord, New Haven, Conn.

Section IV. Dental Physics, Radiography and Metallurgy, Dr. J. P. Buckley, Chicago, Ill.

Section V. Dental Prosthesis, Dr. D. O. M. LeCron, London, England.

Section VI. Orthodontics, Dr. Roscoe A. Day, San Francisco, Calif.

Section VII. Oral Surgery and Surgical Prosthesis, Dr. J. D. Patterson, Kansas City, Mo.

Section VIII. Anaesthesia, General and Local, Dr. Thos. P. Hinman, Atlanta, Ga.

Section IX. Oral Hygiene, Public Instruction and Public Dental Services, Dr. Herbert L. Wheeler, New York City.

Section X. Dental Education, Dr. Henry W. Morgan, Nashville, Tenn.

A list of essayests and clinicians will be published later.

The committee invite the ethical members of the profession of the U. S. A., to become members of the Congress. Membership, which includes admission to the Congress sessions and a copy of the proceedings is $7.50 and for members of their families accompanying them $3.75.

Dr. Herbert L. Wheeler, 160 Fifth Ave., New York City, has been appointed by the committee to arrange for steamship rates, sailing dates, itinerary, etc. Those desiring to attend the Congress, sailing with the American delegation immediately following the meeting of the National Dental Association, Rochester, N. Y., July 10, 1914, are requested to correspond with Dr. Wheeler.

BURTON LEE THORPE, Secretary,
3605 Lindell Blvd., St. Louis, Mo.

COMMITTEE {

TRUMAN W. BROPHY, Chairman.

WM. CARR,

S. H. GUILFORD,

WALDO E. BOARDMAN.

